# Parenting a child with Marfan syndrome: Distress and everyday problems

**DOI:** 10.1002/ajmg.a.61906

**Published:** 2020-10-09

**Authors:** Jessica Warnink‐Kavelaars, Hedy A. van Oers, Lotte Haverman, Annemieke I. Buizer, Mattijs W. Alsem, Raoul H. H. Engelbert, Leonie A. Menke

**Affiliations:** ^1^ Department of Rehabilitation Medicine Amsterdam UMC, University of Amsterdam, Amsterdam Movement Sciences Amsterdam Netherlands; ^2^ Psychosocial Department Emma Children's Hospital, Amsterdam UMC, University of Amsterdam Amsterdam Netherlands; ^3^ Department of Rehabilitation Medicine Amsterdam UMC, Vrije Universiteit Amsterdam, Amsterdam Movement Sciences Amsterdam Netherlands; ^4^ Center of Expertise Urban Vitality University of Applied Sciences, Faculty of Health Amsterdam The Netherlands; ^5^ Department of Pediatrics Emma Children's Hospital, Amsterdam UMC, University of Amsterdam Amsterdam Netherlands

**Keywords:** autosomal dominant, chronic illness, connective tissue disorder, distress, Marfan syndrome, parents

## Abstract

Marfan syndrome (MFS) is a multisystemic, autosomal dominant connective tissue disorder that occurs de novo in 25%. In many families, parent and child(ren) are affected, which may increase distress in parents. To assess distress, 42 mothers (29% MFS) and 25 fathers (60% MFS) of 43 affected children, completed the validated screening‐questionnaire *Distress thermometer for parents of a chronically ill child*, including questions on overall distress (score 0–10; ≥4 denoting “clinical distress”) and everyday problems (score 0–36). Data were compared to 1,134 control‐group‐parents of healthy children. Mothers reported significantly less overall distress (2, 1–4 vs. 3, 1–6; *p* = .049; *r* = −.07) and total everyday problems (3, 0–6 vs. 4, 1–8; *p* = .03; *r* = −.08) compared to control‐group‐mothers. Mothers without MFS reported significantly less overall distress compared to mothers with MFS, both of a child with MFS (1, 0–4 vs. 3.5, 2–5; *p* = .039; *r* = −.17). No significant differences were found between the father‐groups, nor between the group of healthy parents of an affected child living together with an affected partner compared to control‐group‐parents. No differences in percentages of clinical distress were reported between mothers and control‐group‐mothers (33 vs. 42%); fathers and control‐group‐fathers (28 vs. 32%); nor between the other groups. Distress was not associated with the children's MFS characteristics. Concluding, parents of a child with MFS did not show more clinical distress compared to parents of healthy children. However, clinical distress was reported in approximately one‐third and may increase in case of acute medical complications. We advise monitoring distress in parents of a child with MFS to provide targeted support.

## 1 INTRODUCTION

Marfan syndrome (MFS) is an autosomal dominant connective tissue disorder caused by a pathogenic variant in *FBN1* (Loeys et al., 2010) and occurs de novo in a quarter of patients. In many families, both a parent and one or more children are diagnosed with MFS. The estimated prevalence is 1:5,000–1:10,000 (Dietz, 1993) and the diagnosis is based on the revised Ghent criteria (Loeys et al., 2010). Children and adults/parents with MFS need regular medical follow‐up (Hilhorst‐Hofstee, 2013; Rozado, Martin, Pascual, Hernandez‐Vaquero, & Moris, 2017; Tinkle & Saal, 2013) because of the risk of developing medical complications of the cardiovascular‐ (aortic aneurysm, mitral valve prolapse), musculoskeletal‐ and ophthalmic‐ (ectopia lentis, severe myopia) systems (Dietz, 1993; Faivre et al., 2012; Loeys et al., 2010; Sheikhzadeh et al., 2012; Stheneur et al., 2014; Velvin, Bathen, Rand‐Hendriksen, & Geirdal, 2015a, 2016b). Therefore, parents may have extended caregiving responsibilities, both for their child/children with MFS and for themselves or their partner with MFS, which may further increase distress and everyday problems.

In a recent study, we found that parents of a child with MFS reported parental burden caused by high parental caring requirements for their child's medical and psychosocial needs, lack of professional health care support, a limited social life, parental concerns about their child's physical, psychosocial development and fear of high‐risk aortic surgery or early death (Warnink‐Kavelaars et al., 2019). Also, in parents of children with other chronic illnesses, parental functioning was negatively affected (Pinquart, 2013) as well as their participation (Hatzmann, Peek, Heymans, Maurice‐Stam, & Grootenhuis, 2014). Parents suffered from anxiety and depression (van Oers et al., 2014), parenting stress (Cousino & Hazen, 2013) and parental burden (Biber et al., 2019; Jackson, Frydenberg, Liang, Higgins, & Murphy, 2015; Jackson, Higgins, Frydenberg, Liang, & Murphy, 2018). Moreover, parents of a child with cancer (Schepers et al., 2018), home parenteral nutrition (van Oers et al., 2019), mucopolysaccharidosis type III (Conijn, Nijmeijer, van Oers, Wijburg, & Haverman, 2019) inflammatory bowel disease (Diederen, Haverman, Grootenhuis, Benninga, & Kindermann, 2018), Down syndrome (Marchal et al., 2017) and a chronic disease of any type (van Oers, Schepers, Grootenhuis, & Haverman, 2017), screened by the *Distress thermometer for parents of a chronically ill child* (DT‐P) (Haverman, van Oers, Limperg, Hijmans, et al., 2014; Haverman, van Rossum, van Veenendaal, van den Berg, et al., 2013) reported significantly higher distress and/or more often everyday problems compared to control‐group parents. These and other studies also reported significant differences in distress levels of mothers compared to fathers (Conijn et al., 2019; Marchal et al., 2017; Schepers et al., 2018; Sultan, Leclair, Rondeau, Burns, & Abate, 2016; van Oers et al., 2014; van Oers et al., 2019). There is limited knowledge of the distress of parents who have a chronic illness themselves. Some studies reported the adverse effects of chronic illness on parental health‐related quality of life (Hatzmann et al., 2014; Hatzmann, Maurice‐Stam, Heymans, & Grootenhuis, 2009) and a tendency of limited social and family activities for all family members (Janotha, 2011). Studies on distress in parents and parenting a child with a chronic or connective tissue disorder while being affected by the same disorder; as well as studies on distress in healthy parents and caring for an affected partner and an affected child, are even rarer. However, studies reporting on the health‐related effects of MFS in adults on family life, physical activities, psychosocial development, education, work, and reproductive planning provide clues for understanding distress in parents with MFS (Nielsen, Ratiu, Esfandiarei, Chen, & Selamet Tierney, 2019; Peters, Horne, Kong, Francomano, & Biesecker, 2001; Peters, Kong, Hanslo, & Biesecker, 2002; Peters, Kong, Horne, Francomano, & Biesecker, 2001; Speed et al., 2017; Velvin et al., 2015a; Velvin et al., 2016b; Velvin, Bathen, Rand‐Hendriksen, & Geirdal, 2015b, 2016a).

This study aims to assess distress and everyday problems of mothers and fathers without and with MFS, of a child with MFS using the DT‐P. Data are compared to those of control‐group mothers and fathers of a healthy child. Associations will be explored between distress in parents and the presence of MFS characteristics of the child.

## 2 MATERIALS AND METHODS

### 2.1 Participants and procedures

Eligible for inclusion were all mothers and fathers of a child aged 0–18 years, diagnosed with MFS according to the revised Ghent criteria (Loeys et al., 2010), who visited the Amsterdam Expert Center for children with Marfan syndrome and related disorders between June 2017 and May 2019. One week before the annual outpatient visit of their child, the parents were invited by letter to both complete the online DT‐P and questions on sociodemographic characteristics on the KLIK website (www.hetklikt.nu). KLIK is an online Patient‐Reported Outcome Measure (PROM portal) to systematically monitor different aspects of children with various chronic illnesses and their parents over time. Answers to the questionnaires (PROMs) were converted into a KLIK PROfile and discussed during the outpatient visit of their child (Haverman, van Oers, Limperg, Hijmans, et al., 2014; Haverman, van Rossum, van Veenendaal, van den Berg, et al., 2013).

The Medical Ethics Review Committee of the Amsterdam University Medical Centers, Amsterdam, the Netherlands, waived ethical approval under Dutch Law. Written informed consent was obtained from all parents for the reuse of data for research.

### 2.2 Measurements

#### 2.2.1 Sociodemographic characteristics

Parents completed online questions on sociodemographic characteristics including their age, country of birth, educational level, employment status, marital status, number of children living at home, as well as the age, gender and educational level of their child with MFS.

#### 2.2.2 Distress thermometer for parents (DT‐P)

The DT‐P is a validated screening instrument to identify overall distress, clinical distress and everyday problems in parents of a chronically ill child (Haverman, van Oers, Limperg, Houtzager, et al., 2013; van Oers et al., 2017). The DT‐P consists of three parts. First, parents rate their overall distress in the past week on a “thermometer” ranging from 0 (no distress) to 10 (extreme distress) with a thermometer score ≥ 4 indicating clinically relevant distress (further referred to as “clinical distress”). Second, the occurrence of everyday problems is inquired by 36 or 34 problem item yes/no questions (for parents of a child <2 years or ≥ 2 years of age, respectively). There are six everyday problem domain scores: practical, social, emotional, physical, cognitive and parenting. These everyday problem domain scores are based on the number of times a “yes” is filled in for the everyday problem domain items. Third, additional questions inquire (a) perceived support from surroundings, (b) perceived lack of understanding from others concerning their situation, (c) parental chronic illness, (d) the wish to talk to a professional about their situation (yes, maybe or no) (Haverman, van Oers, Limperg, Houtzager, et al., 2013; van Oers et al., 2017). The internal consistency of the DT‐P is acceptable with Cronbach's alphas ranging from 0.52 to 0.89 (van Oers et al., 2017).

#### 2.2.3 Marfan syndrome characteristics in children

The revised Ghent systemic score and the child‐reported pain and fatigue were used to decribe the presence of MFS characteristics in children. Other characteristics that could have been used, for instance, aortic dilatation, lens luxation, foot‐, lens‐, pectus and/or scoliosis surgery, were too infrequently encountered. The revised Ghent systemic score is part of the revised Ghent criteria and is a method of assigning weighted values to the presence of clinical features that are associated with MFS. The score is calculated through the summation of applicable points (0–20). Experienced pain and fatigue of the child were discussed during the outpatient visit, 1 week after filling in the DT‐P, and categorized in "no,” “sometimes,” or “often.” Data were extracted from the child's medical file.

### 2.3 Statistical analyses

Mothers and fathers of a child with MFS were analyzed as separate groups because of reported differences in distress levels (Conijn et al., 2019; Marchal et al., 2017; Schepers et al., 2018; Sultan et al., 2016; van Oers et al., 2014; van Oers et al., 2019). The Statistical Package for Social Sciences (SPSS) version 25.0 for Windows was used for all statistical analyses.

Descriptive analyses were used to describe the sociodemographic characteristics of the mothers, fathers without and with MFS and their children with MFS. Data were compared to those of 671 control‐group mothers and 463 control‐group fathers of a healthy child (van Oers et al., 2017) using independent samples *t*‐tests for numerical data and Chi‐square tests for categorical data. Overall distress score, total everyday problem score and everyday problem domain scores were not distributed normally and so the median (interquartile range: IQR) was reported. Comparisons between groups were performed using Mann–Whitney *U* tests: between (a) mothers of a child with MFS and control‐group mothers; (b) mothers without MFS and mothers with MFS, both of a child with MFS; (c) mothers without MFS of a child with MFS, living together with an affected partner and control‐group mothers; (d) fathers of a child with MFS and control‐group fathers; (e) fathers without MFS and fathers with MFS, both of a child with MFS; (f) fathers without MFS of a child with MFS, living together with an affected partner and control‐group fathers. Effect sizes (*r*) were calculated. The clinical distress score, everyday problem items and the additional questions were analyzed with Chi‐square/Fisher's exact tests; odds ratios (OR) and confidence intervals (CI) were calculated. Following the previous DT‐P studies, problem domain items were also analyzed for exploration and therefore, we did not correct for multiple testing. Correlation analyses (Spearman's rho) were used to explore associations between distress in parents and the presence of MFS characteristics of the child using the revised Ghent systemic score and the child‐reported pain and/or fatigue.

## 3 RESULTS

### 3.1 Sociodemographic characteristics

In total, 42 mothers (29% with MFS) and 25 fathers (60% with MFS) of 43 children with MFS completed the DT‐P (response rate 57%). Of the parents without MFS of a child with MFS, 14 mothers and 7 fathers lived together with an affected partner. No differences were found between the socio‐demographic characteristics of mothers, fathers of a child with MFS and their children with MFS and control‐group mothers, control‐group fathers and their healthy children (Table 1), and between mothers without MFS and mothers with MFS, both of a child with MFS, nor between fathers without MFS and fathers with MFS, both of a child with MFS (data not shown).

**TABLE 1:** Sociodemographic characteristics of parents of a child with MFS; control‐group parents of a healthy child; children with MFS; and control‐group healthy children

Parents	Mothers			Fathers		
	Mothers of a child with MFS (*N* = 42)	Control‐group mothers of healthy children (*N* = 671)	*p* value	Fathers of a child with MFS (*N* = 25)	Control‐group fathers of healthy children (*N* = 463)	*p* value
Age in years, mean (*SD*), range	40.4 (6.8), 25.7–51.9	38.8 (6.4), 18.1–63.3	.096	42.0 (7.2), 28.0–52.6	41.7 (7.4), 26.2–75.3	.835
Born in the Netherlands, *n* (%)	38 (90.5)	647 (96.6)	.068^a^	23 (92.0)	442 (95.5)	.332^a^
Educational level, *n* (%)^b^			.567			.095
Low	3 (7.3)	88 (13.1)		0	72 (15.6)	
Intermediate	17 (41.5)	300 (44.7)		10 (40.0)	193 (41.7)	
High	21 (51.2)	281 (41.9)		15 (60.0)	190 (41.0)^c^	
Paid employment, *n* (%)	32 (76.2)	545 (81.2)	.688	21 (84.0)	433 (93.5)^d^	.141
Marital status, *n* (%)			.991			.135
Married/living together	38 (90.5)	604 (90.0)		24 (96.0)	449 (97.0)	
Single/separated	4 (9.5)	64 (9.5)		1 (4.0)	13 (2.8)	
Widow	0	2 (0.3)		0	1 (0.2)	
Children living at home, *n* (%)			.876			.917
1	10 (23.8)	138 (20.6)		5 (20.0)	82 (17.7)	
2	23 (54.8)	378 (56.3)		15 (60.0)	274 (59.2)	
≥3	9 (21.4)	155 (23.1)		5 (20.0)	107 (23.1)	
Parental diagnosis of MFS, *n* (%)						
Yes	12 (28.6)	N/A		15 (60.0)	N/A	
No	27 (64.3)	N/A		10 (40.0)	N/A	
Not tested	3 (7.1)			0		

Children			
	Children with MFS (*N* = 43)	Control‐group healthy children (*N* = 1,134)	*p* value
Age in years, mean (*SD*), range	8.9 (4.7), 0.4–17.1	7.5 (5.4) 0.1–19.0	.109
Female gender (%)	19 (44.2)	551 (48.6)	.571
Educational level			.150
None (not yet started), *n* (%)	3 (7.0)	184 (16.2)	
Regular day‐care, *n* (%)	4 (9.3)	197 (17.4)	
Regular primary school, *n* (%)	21 (48.8)	478 (42.2)	
Special primary school, *n* (%)	1 (2.3)	5 (0.4)	
Regular secondary school, *n* (%)	12 (27.9)	206 (18.2)	
Special secondary school, *n* (%)	0 (0)	4 (0.4)	
Post‐secondary school, *n* (%)	2 (4.7)	60 (5.3)	
Having a parent with MFS, *n* (%)	31 (72.1)	N/A	
Revised Ghent score, median (*SD*), range	6.7 (3.1), 1–13	N/A	
Child reported pain sometimes‐/often, *n* (%)	10 (23.3)	N/A	
Child reported fatigue sometimes‐/often, *n* (%)	19 (44.2)	N/A	

Abbreviations: MFS, Marfan syndrome; *p*, probability; *n*, number; N/A, not applicable; High, higher vocational education, university; Intermediate: middle vocational education, higher secondary education, pre‐university education; Low: primary education, lower vocational education, lower or middle general secondary education.

^a^ Fishers Exact (<*N* = 5 in one cell).

^b^ One missing.

^c^ Eight missing.

^d^ Two missing.

### 3.2 Marfan syndrome characteristics in the children

The diagnosis MFS was molecularly confirmed in 42 of the 43 children. The mean revised Ghent systemic score of the children was 6.7 (*SD*, 3.1; range, 1–13; Table 1). “Sometimes/often” pain was reported in 23% and “sometimes/often” fatigue was reported in 44% of children with MFS (Table 1).

### 3.3 Overall distress

Overall distress scores are shown in Table 2. The median overall distress score (IQR) of mothers of a child with MFS was significantly lower compared to control‐group mothers (2, 1–4 vs. 3, 1–6; *p* = .049; *r* = −.07). Mothers without MFS reported significantly less overall distress compared to mothers with MFS, both of a child with MFS (1, 0–4 vs. 3.5, 2–5; *p* = .039; *r* = −.17). No significant differences in overall distress were found between the other groups.

**TABLE 2:** DT‐P overall and clinical distress score, problem domain scores and additional question scores of mothers and fathers of a child with MFS compared to control‐group mothers and fathers of healthy children

Parents	Mothers					Fathers				
	Mothers of a child with MFS (*N* = 42)	Control‐group mothers of healthy children (*N* = 671)	*p* value	*r*/OR	*z*‐score/95% CI	Fathers of a child with MFS (*N* = 25)	Control‐group fathers of healthy children (*N* = 463)	*p* value	*r* /OR	*z*‐score/95% CI
Distress score										
Overall, median (IQR)	2 (1–4)	3 (1–6)	**.049**	−.07	*z* = −1.966	2 (1–6)	2 (1–5)	.68	−.02	*z* = −0.418
Clinical %	33.3	42.3	.252	.68	0.35–2.84	28.0	32.2	.662	.82	0.33–2.0
*Total problem domain score*, median (IQR)^a^	3 (0–6)	4 (1–8)	**.032**	−.08	*z* = −2.148	1.5 (0–6)	2 (1–6)	.184	−.06	*z* = 1.330
Problem domains										
Practical problems, median (IQR)	0.5 (0–2)	1 (0–2)	**.037**	−.08	*z* = −2.084	0 (0–1)	0 (0–1)	.880	−.01	*z* = −0.151
Social problems, median (IQR)	0 (0–0)	0 (0–1)	**.032**	−.08	*z* = −2.142	0 (0–0)	0 (0–0)	.850	−.01	*z* = −0.189
Emotional problems, median (IQR)	0 (0–2.25)	1 (0–3)	.257	−.04	*z* = −1.133	0 (0–1.5)	0 (0–2)	.372	−.04	*z* = −0.892
Physical problems, median (IQR)	0.5 (0–2)	2 (0–3)	**.016**	−.09	*z* = −2.419	1 (0–2)	1 (0–2)	.839	−.01	*z* = −0.203
Cognitive problems, median (IQR)	0 (0–0)	0 (0–1)	.102	−.06	*z* = −1.637	0 (0–0)	0 (0–0)	.655	−.02	*z* = −0.447
Parenting problems child ≥2 years, median (IQR)^b^	0 (0–0)	0 (0–0)	.086	−.06	*z* = −1.718	0 (0–0)	0 (0–0)	.518	−.03	*z* = −0.646
Parenting problems child <2 years^c^										
Additional questions support from others										
Experiencing enough support from others, and environment %	92.9	92.1	1.00^d^	1.11	0.33–3.70	92.0	93.3	.683^d^	.82	0.19–3.70
Experiencing a lack of understanding from others (%)	11.9	11.3	.909	1.05	0.40–3.18	12.0	10.2	.733^d^	1.20	0.35–4.17
Having a chronic illness themselves (%)	40.5	20.3	**.002**	2.70	1.40–5.0	64.0	14.0	**.000**	11.11	4.55–25.0
Would like to talk to a professional about situation—Yes/Maybe (%)	19.0	17.1	.751	1.14	.51–2.50	24.0	12.5	.098	2.22	0.84–5.88

*Note:* Significant differences at *p* < .05 are presented in bold; distress and domain scores: numerical data > not normal distributed > Mann–Whitney *U* tests with *Z* score (*z*) and effect size (*r*); binary data > Chi‐square tests with odds ratio (OR) and confidence interval (CI).

Abbreviations: IQR, interquartile range; MFS, Marfan syndrome; *p* value, probability value; OR, odds ratio; *r*, effect size; *n*, number.

^a^ Total problem score = the sum of item scores (yes = 1, no = 0) within 6 problem domains (practical, social, emotional, physical, cognitive and parenting).

^b^ *N* = 41 MFS mothers, *N* = 560 reference mothers, *N* = 24 MFS fathers, *N* = 370 reference fathers.

^c^ *n* = 1, no calculations possible.

^d^ Fisher's Exact (<*N* = 5 in one cell).

### 3.4 Clinical distress

Clinical distress scores are shown in Table 2. No differences in percentages of clinical distress were found between mothers compared to control‐group mothers (33 vs. 42%); mothers without MFS compared to mothers with MFS, both of a child with MFS (26 vs. 50%); mothers without MFS of a child with MFS, living together with an affected partner, compared to control‐group mothers (29 vs. 42%); fathers of a child with MFS compared to control‐group fathers (28 vs. 32%); fathers without MFS compared to fathers with MFS, both of a child with MFS (30 vs. 27%); fathers without MFS of a child with MFS, living together with an affected partner, compared to control‐group fathers (29 vs. 32%).

### 3.5 Everyday problems

Total and everyday problem domain scores are shown in Table 3.

**TABLE 3:** DT‐P everyday problem‐item scores of mothers and fathers of a child with MFS compared to control‐group mothers and fathers of healthy children

Parents	Mothers					Fathers				
	Mothers of a child with MFS (*N* = 42)	Control‐group mothers of healthy children (*N* = 671)	*p*	OR	95% CI	Fathers of a child with MFS (*N* = 25)	Control‐group fathers of healthy children (*N* = 463)	*p*	OR	95% CI
Practical problems										
Housing (%)	4.8	5.5	1.00^a^	0.85	0.20–3.70	8.0	3.7	.253^a^	2.27	0.50–10
Work/study (%)	26.2	25.3	.902	1.04	0.52–2.13	28.0	25.9	.817	1.11	0.45–2.70
Finances/insurance (%)	0.0	16.7	**.001** ^a^	^a^		12.0	14.5	1.00^a^	0.80	0.23–2.77
Housekeeping (%)	11.9	21.6	.134	0.49	0.19–1.27	12.0	12.1	1.00^a^	0.99	0.29–3.44
Transport (%)	4.8	4.6	1.00^a^	1.03	0.24–4.55	4.0	3.9	1.00^a^	1.03	0.13–8.33
Child care/child supervision (%)	4.8	10.1	.419	0.44	0.10–1.89	4.0	5.4	1.00^a^	0.73	0.09–5.55
Leisure activities/relaxing (%)	19.0	22.4	.617	0.82	0.37–1.79	20.0	14.9	.489	1.43	0.52–4.00
Social problems										
Dealing with (ex)partner (%)	2.4	12.4	**.049** ^a^	0.17	0.02–0.92	12.0	11.7	1.00^a^	1.03	0.30–3.57
Dealing with family (%)	4.8	10.9	.300^a^	0.41	0.10–1.72	4.0	6.7	1.00^a^	0.58	0.08–4.35
Dealing with friends (%)	4.8	3.7	.669^a^	1.30	0.30–5.56	12.0	1.5	**.011** ^a^	9.09	2.12–33.33
Interacting with your child(ren) (%)	4.8	11.8	.213^a^	0.37	0.09–1.59	12.0	7.8	.440^a^	1.61	0.46–5.56
Emotional problems										
Controlling emotions (%)	19.0	27.4	.235	0.62	0.28–1.37	12.0	11.9	1.00^a^	1.01	0.29–3.45
Self‐confidence (%)	9.5	22.7	.053^a^	0.36	0.13–1.02	12.0	12.7	1.00^a^	0.93	0.27–3.23
Fears (%)	16.7	10.7	.156	1.67	0.71–3.85	12.0	6.5	.234^a^	1.96	0.56–7.14
Depression (%)	21.4	31.9	.151	0.58	0.27–1.23	24.0	22.2	.838	1.10	0.43–2.86
Feeling tense or nervous (%)	38.1	36.1	.791	1.09	0.57–2.08	24.0	26.3	.795	0.88	0.35–2.27
Loneliness (%)	2.4	7.7	.356^a^	0.29	0.04–2.17	12.0	3.7	.076^a^	3.57	0.97–12.50
Feelings of guilt (%)	9.5	17.4	.287^a^	0.50	0.17–1.43	12.0	7.3	.425^a^	1.72	0.49–5.88
Use of substances (e.g., alcohol, drugs and/or medication) (%)	2.4	2.7	1.00^a^	0.88	0.12–6.66	0.0	3.0	1.00^a^	^b^	
Intrusive/recurrent thoughts about a specific event (%)	21.4	20.4	.875	1.06	0.50–2.27	16.0	13.8	.766^a^	1.19	0.40–3.57
Physical problems										
Eating (%)	4.8	12.4	.215^a^	0.44	0.08–1.49	16.0	4.8	.**037** ^a^	3.85	1.20–12.50
Weight (%)	19.0	26.2	.302	0.66	0.30–1.45	4.0	16.6	.155^a^	0.21	0.03–1.56
Sleep (%)	26.2	29.7	.633	0.84	0.41–1.69	12.0	21.4	.322^a^	0.50	0.15–1.69
Fatigue (%)	35.7	55.7	**.011**	0.44	0.23–.84	40.0	44.1	.690	0.85	0.37–1.92
Out of shape/condition (%)	11.9	20.9	.162	0.51	0.20–1.33	24.0	19.0	.537	1.35	0.52–3.45
Pain (%)	19.0	24.3	.440	0.74	0.33–1.61	16.0	18.1	1.00^a^	0.86	0.29–2.56
Sexuality (%)	2.4	10.6	.111^a^	0.21	0.03–1.52	16.0	8.9	.274^a^	1.96	0.64–5.88
Cognitive problems										
Concentration (%)	7.1	17.9	.091^a^	0.35	0.12–1.16	20.0	11.2	.184	1.96	0.71–5.56
Memory (%)	14.3	22.4	.220	0.58	0.24–1.41	20.0	13.6	.369	1.59	0.57–4.35
Parenting problems ≥2										
Dealing with your child (%)	4.9	10.9	.297^a^	0.42	0.10–1.79	4.2	9.7	.714^a^	0.40	0.05–3.03
Dealing with the feelings of your child (%)	7.3	9.3	1.00^a^	0.77	0.23–2.56	0.0	8.6	.242^a^	^b^	
Talking about the disease/consequences with your child (%)	0.0	3.0	.621^a^	^b^		0.0	2.7	1.00^a^	^b^	
Independence of your child (%)	2.4	7.5	.348^a^	0.31	0.04–2.27	8.3	7.6	.703^a^	1.11	0.25–5.00
Following advice about treatment/giving medication (%)	0.0	3.4	.633^a^	^b^		4.2	3.0	.535^a^	1.41	0.18–11.11

*Note:* Significant differences at *p* < .05 are presented in bold; item scores: Chi‐square tests with OR and 95% CI.

^a^ Fisher's Exact (<*N* = 5 in one cell).

^b^ No calculation possible due to *n* = 0 in one cell.

Mothers of a child with MFS reported a significantly lower median (IQR) total everyday problem domain score compared to control‐group mothers (3, 0–6 vs. 4, 1–8; *p* = .03; *r* = −.08), with significantly lower scores for the practical problem domain (0.5, 0–2 vs. 1, 0–2; *p* = .037; *r* = −.08); social problem domain (0, 0–0, vs. 0, 0–1; *p* = .032; *r* = −.08) and physical problem domain (0.5, 0–2 vs. 2, 0–3: *p* = .016, *r* = −.09). No significant differences in total and everyday problem domain scores were found between the other groups.

### 3.6 Everyday problem items

Everyday problem items are shown in Table 3.

When looking at the everyday problem items within the 6 problem domains, mothers of a child with MFS reported significantly less often everyday problems on the items finances (0 vs. 16.7%, *p* = .001, *n* = 0 in a cell, no OR calculation possible); dealing with (ex)partner (2.4 vs. 12.4%, *p* = .049, OR = .17, 95% CI .02–.92) and fatigue (35.7 vs. 55.7%, *p* = .01, OR = .44, 95% CI .23–.84), compared to control‐group mothers. Mothers without MFS of a child with MFS, living together with an affected partner, reported significantly more often everyday problems on the item fears compared to control‐mothers (28.6 vs. 10.7%, *p* = .035, OR = 3.3, 95% CI 1.02–10.89). Fathers of a child with MFS reported significantly more often everyday problems on the items dealing with friends (12 vs. 1.5%, *p* = .01, OR = 9.09, 95% CI 2.12–33.33) and eating (16 vs. 4.8%, *p* = .037, OR = 3.85, 95% CI 1.20–12.50), compared to control‐group fathers. Fathers without MFS of a child with MFS, living together with an affected partner, reported significantly more often everyday problems on the items dealing with friends (14.3 vs. 1.5%, *p* = .02, OR = 8.7, 95% CI .95–80.30) and interacting with your child(ren) (28.6 vs. 7.7%, *p* = .043, OR = 4.8, 95% CI .95–25.60) compared to control‐group fathers. No significant differences in the everyday problem items were found between the other groups.

### 3.7 Support from others

Mothers and fathers without and with MFS of a child with MFS, living together with a healthy or an affected partner did not differ significantly from control‐group parents with respect to experiencing to receive enough support from surroundings, experiencing a lack of understanding from others and the wish to talk with a professional about their situation (Table 3). Both mothers and fathers of a child with MFS indicated more often to have a chronic illness than parents of a healthy child (40 vs. 20%, *p* = .002, OR = 2.7, 95% CI 1.40–5.0; 64 vs. 14%, *p* = .000, OR = 11.11, 95% CI 4.55–25.0, Table 3).

### 3.8 Associations of distress and Marfan syndrome characteristics of children

There were no significant associations between distress on the one side, and the revised Ghent systemic score of the child, the child‐reported pain and/or fatigue on the other side.

## 4 DISCUSSION

This study is the first quantitative study reporting on distress and everyday problems in mothers and fathers without and with MFS parenting a child with MFS. Surprisingly, parents of a child with MFS did not show more signs of clinical distress than parents of healthy children. The total group of mothers of a child with MFS even reported significantly lower overall distress and everyday problems compared to control‐group mothers, albeit with small effect sizes.

This was an unexpected finding given the well‐known risk of (acute) medical MFS related complications (Dietz, 1993; Faivre et al., 2012; Loeys et al., 2010; Sheikhzadeh et al., 2012; Stheneur et al., 2014; Velvin et al., 2015a, 2016b), the need for regular medical follow up (Hilhorst‐Hofstee, 2013; Rozado et al., 2017; Tinkle & Saal, 2013) for both children and also for the parent with MFS, and the perceived significant impact of MFS on daily (physical) functioning of children, parents and the family (Nielsen et al., 2019; Peters, Horne, et al., 2001; Peters et al., 2002; Peters, Kong, et al., 2001; Speed et al., 2017; Velvin et al., 2015a, 2015b, 2016a, 2016b; Warnink‐Kavelaars, Beelen, Dekker, et al., 2019; Warnink‐Kavelaars et al., 2019). Parents of children with a variety of other chronic diseases have been shown to often suffer from anxiety and depression (van Oers et al., 2014), parenting stress (Cousino & Hazen, 2013) and parental burden (Biber et al., 2019; Jackson et al., 2015; Jackson et al., 2018). In previous studies in which parental distress was measured by the DT‐P compared to control‐group parents, high overall distress and everyday problems were found in parents of children with cancer (Schepers et al., 2018), mucopolysaccharidosis type III (Conijn et al., 2019), and in children needing home parenteral nutrition (van Oers et al., 2019). In parents of children with Inflammatory Bowel Disease (Diederen et al., 2018), a worsening disease course was directly associated with increased distress. In MFS, however, the clinical features evolve during life, and high‐risk complications or surgery, are only infrequently encountered during childhood. The low level of medical emergencies requiring hospital visits or hospitalization in MFS in childhood may partly explain why we did not find elevated distress nor any association between distress and the child's revised Ghent systemic score, child‐reported pain and/or fatigue. However, medical professionals should be aware that whenever acute medical complications arise, for example, lens luxation, pneumothorax, aortic rupture, musculoskeletal surgery or other surgery in a child or a parent with MFS, distress levels in parents might become clinically relevant and should be addressed accordingly.

Another hypothesis for the unexpected results of our study might be that the parents had developed strong coping strategies. The term “coping” is defined as “the thoughts and behaviors used to manage the internal and external demands of situations that are appraised as stressful, so that it is possible to live and deal with stressful situations and reduce internal and external conflicts and demands” (Folkman & Lazarus, 1980). This is endorsed by a review reporting on psychosocial factors in adults with MFS; despite the psychologically distressing aspects of the diagnosis MFS, most patients were able to manage their stressors and exhibited a higher than average life satisfaction because of efficient coping and reliance on self‐efficacy (Nielsen et al., 2019). In our recent qualitative paper, adolescents with MFS also described positive coping strategies as seeking social support, having a humorous and relaxed outlook on life, reappraising their disease and disability in a positive light, pursuing a healthy lifestyle, and trying to plan their activities well to handle the impact of MFS on their physical and psychosocial functioning (Warnink‐Kavelaars, Beelen, Goedhart, et al., 2019). These adolescents may have copied these strong coping strategies from their parents or the parents may have adopted these strategies from their child.

Little is known about the impact on distress and everyday problems in parents, of parenting a child with a connective tissue disorder (e.g., MFS, Ehlers Danlos, Loeys Dietz syndrome) and being affected by the same disorder or caring for an affected partner and an affected child. In our study, although not significantly different, mothers with MFS tended to report higher clinical distress (50%) compared to control‐group mothers (42%). It is known that in adults, MFS negatively affects family life, physical activities, psychosocial development, education, work, and reproductive planning (Nielsen et al., 2019; Peters, Horne, et al., 2001; Peters et al., 2002; Peters, Kong, et al., 2001; Velvin et al., 2015a, 2016b). Furthermore having a chronic illness as a parent adversely affects parental health‐related quality of life (Hatzmann et al., 2009; Hatzmann et al., 2014). In our study, healthy parents of a child with MFS, living together with an affected partner, did not show more signs of clinical distress compared to control‐group parents of healthy children. However, these mothers reported significantly more often everyday problems on the item fears and fathers reported more often everyday problems on the items dealing with friends and interacting with your child(ren). Because of the negative impact of MFS in adults, for example, personal and family life, medical professionals should be extra alert for distress in parents of families with both a child and a parent with MFS.

Our study has some limitations. First, the sample size might have been too small to find more subtle differences between the groups. Second, all parents were recruited from the Amsterdam Expert Center for children with Marfan syndrome and related disorders. Third, not for every child both parents filled in the questionnaire. One may argue that the one parent with the least problems of the two was more likely to fill in the questionnaire. Also, the DT‐P is linked to the child's hospital visit and asks questions concerning distress in the past week. Parents with MFS with medical complications themselves, busy family schedules, other problems or elevated distress might have canceled the appointment. Therefore, the data may underestimate the distress and everyday problems.

In conclusion, parents of a child with MFS did not show more clinical signs of distress compared to parents of healthy children. Mothers of a child with MFS even reported less overall distress and total everyday problems screened by the DT‐P. The distress in parents was not associated with the children's revised Ghent systemic score, child‐reported pain and/or fatigue.

However, clinical distress was reported in approximately one‐third of parents and may further increase in case of acute medical complications in the child or parent with MFS. We, therefore, advise monitoring distress in parents of a child with MFS so that targeted support can be provided whenever indicated.

## AUTHOR CONTRIBUTIONS

Jessica Warnink‐Kavelaars, Hedy A. van Oers and Leonie A. Menke participated in the study design, data collection and analysis, and the writing of the report. Jessica Warnink‐Kavelaars, Hedy A. van Oers and Leonie A. Menke had complete access to the study data that supported the publication. All authors revised the manuscript critically, approved the final version, and agreed to its submission for publication.

The listed authors all met the appropriate authorship criteria. No qualified authors were omitted from the list. All contributors have been appropriately acknowledged, and all authors and contributors have approved the acknowledgment of their contributions.

## CONFLICT OF INTEREST STATEMENT

The authors declare that they have no conflict of interest.

## Data Availability

All data are available on request from the corresponding author.
